# Determination of Residual Epichlorohydrin in Sevelamer Hydrochloride by Static Headspace Gas Chromatography with Flame Ionization Detection

**DOI:** 10.3797/scipharm.1007-20

**Published:** 2010-09-26

**Authors:** Kaliaperumal Karthikeyan, Govindasamy T. Arularasu, Perumalsamy Devaraj, Karnam Chandrasekara Pillai

**Affiliations:** 1 Analytical Development, Shasun Research Center, 27, Vandalur Kelambakkam Road, Keelakottaiyur, Chennai 600048, India; 2 Department of Physical Chemistry, University of Madras, Guindy Campus, Chennai 600025, India

**Keywords:** Genotoxin, Carcinogen, Drug substance, Polymeric phosphate binder, GC

## Abstract

A sensitive static headspace gas chromatographic method was developed and validated for the determination of residual epichlorohydrin (ECH) in sevelamer hydrochloride (SVH) drug substance. This method utilized a Phenomenex Zebron ZB-WAX GC column, helium as carrier gas with flame ionization detection. The critical experimental parameters, such as, headspace vial incubation time and incubation temperature were studied and optimized. The method was validated as per United States Pharmacopoeia (USP) and International Conference on Harmonization (ICH) guidelines in terms of detection limit (DL), quantitation limit (QL), linearity, precision, accuracy, specificity and robustness. A linear range from 0.30 to 10 μg/mL was obtained with the coefficient of determination (r^2^) 0.999. The DL and QL of ECH were 0.09 μg/mL and 0.30 μg/mL, respectively. The recovery obtained for ECH was between 91.7 and 96.6%. Also, the specificity of the method was proved through gas chromatography mass spectrometry (GC-MS). This method was applied successfully to determine the content of residual ECH in SVH bulk drug.

## Introduction

**Fig. 1.**
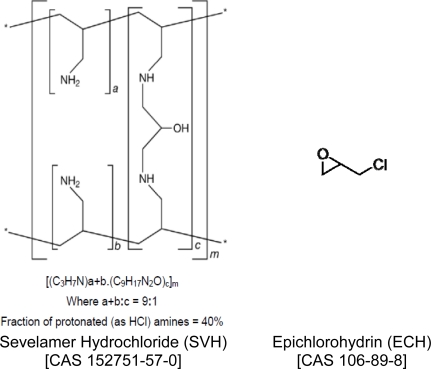
Chemical structure of Sevelamer HCl and Epichlorohydrin

Sevelamer hydrochloride (SVH) drug substance is intended for oral administration in the treatment of hyperphosphatemia. SVH acts as a polymeric phosphate binder and it has been shown to decrease serum phosphorus concentrations in patients with end-stage renal disease. SVH is known chemically as poly(allylamine-co-*N*,*N*’-diallyl-1,3-diamino-2-hydroxypropane hydrochloride) [1]. It is poly(allylamine hydrochloride) crosslinked with epichlorohydrin in which forty percent of the amines are protonated. The primary amine groups in the structure (Fig. 1.) are derived directly from poly(allylamine hydrochloride), and the crosslinking groups consist of two secondary amine groups derived from poly (allylamine hydrochloride) and one molecule of epichlorohydrin (ECH). ECH, a bifunctional alkylating agent, chemically described as 2-(chloromethyl)oxirane, belongs to a class of epoxide compounds that are considered to be “probably carcinogenic to humans” based on structure activity relationship [2]. Because of the known carcinogenicity and structural alert for genotoxicity, the presence of residual ECH in SVH drug substance must be controlled as per European Medicines Agency (EMEA), International Conference on Harmonization (ICH) and Food and Drug Administration (FDA) guidelines [3–7]. EMEA and FDA guidelines proposed the use of the “threshold of toxicological concern” (TTC) concept for the limit of genotoxic / carcinogenic impurities. The TTC refers to a threshold exposure level to compounds that do not pose a significant risk for carcinogenicity / genotoxicity or other toxic effects; and an exposure level of 1.5 μg per day (TTC) for genotoxic impurity is considered to be associated with an acceptable risk for most pharmaceuticals [8, 9]. The concentration limit, in ppm, of genotoxic impurity in drug substance, is the ratio of TTC in μg per day to the expected dose of drug substance in gram per day. Considering the recommended dose of >2.4g SVH per day, ECH must be limited to less than 0.6 μg/g in the drug substance, necessitating the development of sensitive, accurate and robust analytical method.

SVH is not official in any pharmacopoeia; however, few reports are available for ECH estimation [10–14]. A GC-FID method was reported for the determination of ECH in workplace atmosphere [10]. Trace analysis of ECH in water samples were carried out by GC with electron capture detection (ECD) [11]. Also, ECH content in drinking water was determined as its sulfonate derivative by ion-chromatography with conductivity detection [12]. Lasa et. al published a headspace solid-phase micro extraction (SPME) procedure followed by GC analysis for ECH estimation in water [13]. GC-MS method for monitoring exposure to ECH by means of quantitative analysis of *N*-(2,3-dihydroxypropyl)valine adduct in hemoglobin was reported [14]. The published methods evaluated ECH, mostly on clean sample matrices, such as, water and air. Additionally, these methods [10–14] utilized derivatization procedures, adduct analysis, SPME fibers, mass and electrochemical detectors. The complexities and limitations of the existing methods and no method reported so far, for the determination of ECH in polymeric drug substance (SVH), prompted to develop a new method.

The present investigation was, therefore, initiated with the objective to develop a simple and sensitive analytical method for the ECH estimation in SVH drug substance utilizing static headspace (HS) GC with FID. An important and critical aspect about SVH is related to its solubility. Being a polymeric material, SVH is poorly soluble in most of the commonly used organic and aqueous solvents and hence trace level ECH determination in SVH sample by a direct and conventional GC method is practically difficult. Thus, the more versatile and sensitive static HS-GC method, which is widely used for the estimation of organic volatile analytes / residual solvents / reagents in drug compounds was selected. The prime advantage of static HS-GC is its application to drug substances / products that have low solubility or generate degradation products or thermally decompose on column and interfere with the volatile analyte peaks when injected directly [15–17]. Additionally, the availability of high level of automation in the static HS instrumentation has extended its application potentiality for ECH estimation. Selection of solvents used for extraction, HS vial incubation time, incubation temperature and the types of GC column were found to play a vital role in the sensitivity and ruggedness of the developed method. The proposed HS-GC method with FID has been validated using ICH and USP [18, 19] guidelines as references.

## Results and Discussion

### Development of Chromatographic Method

Because of the poor solubility of SVH, preliminary experiments were conducted to optimize the suitable solvent for HS extraction. As SVH is a known hydrophilic polymer having the tendency to swell with water [1], utilization of water as solvent was not considered. Several commonly used organic solvents, such as, DMF, DMSO, NMP and DMAc were studied. Among the four organic solvents, DMSO was selected because it did not have interference peaks and the recovery of ECH was good, whereas, DMF and DMAc had interference peaks at the retention of ECH. Problems with poor peak shape and recovery were encountered with NMP. Different types of GC columns, such as, DB624 (6% cyanopropylphenyl – 94% dimethyl polysiloxane), DB1 (100% dimethyl polysiloxane), and ZBWAX (polyethylene glycol) were screened. ZBWAX column yielded a better peak efficiency (shape) and acceptable recovery compared to other columns.

**Fig. 2.**
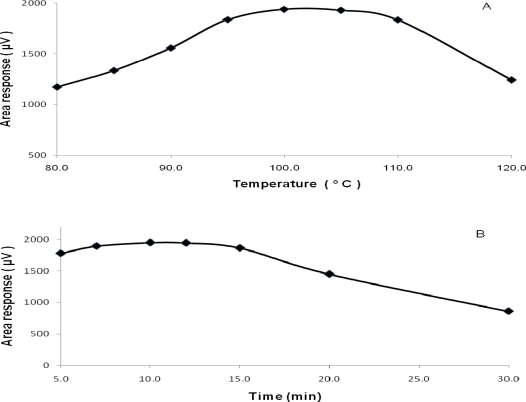
Effect of variation in (A) incubation temperature and (B) incubation time on the area response of ECH

As a part of method development and robustness evaluation, the following parameters, such as, HS incubation temperature, incubation time, flow rate and column oven temperature, were studied to achieve high sensitivity and good precision. From the investigation, HS incubation time and temperature were found to be very critical. The effect of variation in HS incubation time and temperature on the area response of ECH was represented graphically in Fig. 2. When the incubation temperature was studied from 80 °C to 120 °C, there was gradual increase in the area response from 80 °C, reached maximum at 100 °C and it was stable up to 105 °C, then, it decreased significantly at 120 °C. The reason for the lower area at 80 °C could be due to insufficient temperature to bring ECH to the gaseous head space and decrease in the area after 105 °C might be due to the interaction / reaction of ECH with drug substance. Similarly, when incubation time was studied from 5 min to 30 min, peak response of ECH was maximum and almost same between 7–15 min but there was a decrease in the area at 30 min. The lower response at 5 min incubation could be due to inadequate equilibration and sudden decrease in area after 15 min might be due to interaction / reaction of ECH with drug substance. Since, ECH was used as a cross-linker in the preparation of SVH drug, there is possibility for its reaction with drug at higher temperature and prolonged incubation time. Hence, 100 °C incubation temperature and 10 min incubation time were finalized in the proposed method. The variation in flow rate and column oven temperature had not influenced significantly the area response and results. Helium was preferred as carrier gas over nitrogen to obtain a stable and consistent baseline. Since, ECH content in SVH sample was less than the detection limit (<0.09 μg/mL), SVH sample spiked with ECH at 1.5 μg/g level was used in all the developmental and validation studies. Typical retention time of ECH was 10 min. The retention time of ECH peak in spiked sample preparation was found to be consistent with standard in all the studies.

## Method validation

### Specificity

**Fig. 3.**
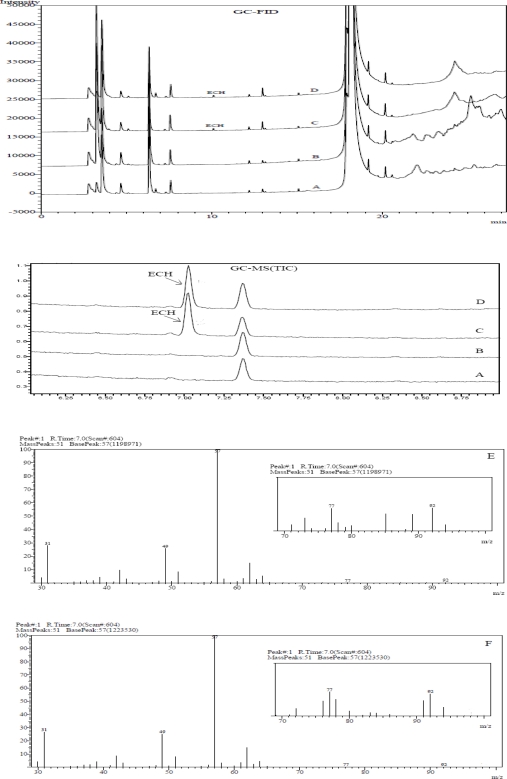
Overlaid chromatograms of GC-FID and GC-MS of (A) Blank, (B) SVH sample preparation, (C) ECH standard preparation and (D) SVH sample spiked with ECH; Mass spectrum of (E) ECH standard preparation and (F) SVH sample spiked with ECH.

To establish the specificity of the method, SVH sample and ECH spiked SVH sample preparations were subjected to hyphenated HS-GC-MS in addition to HS-GC-FI detection. No interference was found during the FID at the retention of ECH as illustrated in Fig. 3. The identity and specificity of ECH peak was further demonstrated through GC-MS. The total ion chromatogram (TIC) and mass spectrum of ECH and SVH spiked with ECH are presented in Fig. 3. The mass spectrum of ECH showed a parent peak at m/z 92 corresponding to the molecular formula C_3_H_5_ClO. Also, it contain peaks corresponding to major fragments at m/z values 57 (base peak) and 49. The mass fragments, including base peak of ECH standard and SVH sample spiked with ECH were found comparable with National Institute of Standards Technology (NIST) mass spectral library for ECH. From the GC-MS and FID data, it could be concluded that the proposed method is specific for ECH determination.

### Repeatability

ECH standard solution (0.75 μg/mL) was injected in six replicates. The RSD (n=6) value obtained for the area response and retention time of ECH was 3.0% and 0.03%, respectively.

### Linearity

The linearity was evaluated by measuring area response for ECH over the range of 0.30 to 10 μg/mL relative to sample concentration (500 mg/mL). Nine concentrations (n=9) were prepared across the range and injected each in triplicate. The mean (n=3) area calculated was plotted against the concentration. The coefficient of determination (r^2^) obtained for ECH was 0.999. The slope of regression line and y-intercept was 1232 and −87.6, respectively (Tab. 1.).

### Accuracy

Accuracy of the method was validated through recovery experiments by spiking known amount of ECH at 0.30, 0.75, 1.5 and 1.8 μg/mL with SVH relative to sample concentration (500 mg/mL). Each preparation was analyzed in triplicate (n=3) and percent recovery was calculated. The recovery was found (Tab. 1.) to be between 91.7 and 96.6% with the RSD of 8.4%

### Detection (DL) and Quantitation (QL) Limit

The DL and QL for ECH were determined by signal-to-noise ratio (S/N) method. The minimum concentration at 3:1 S/N was considered as DL and the concentration at 10:1 S/N was established as QL. The DL and QL obtained for ECH was 0.045 and 0.15 μg/mL, respectively, which corresponded to 0.09 and 0.30 μg/mL, relative to sample concentration (500 mg/mL). A solution containing ECH was prepared around its QL concentration and injected in six replicates. The RSD (n=6) value obtained for the area of ECH at QL was 7.2%. (Tab. 1.)

### Stability of Analyte Solution

The stability of ECH in solution was studied by measuring the area response of standard solution (stored at 25 ± 2°C) injected over a period of 48 h. The RSD (n= 7) value obtained for the area response of ECH was 3.3 %.

### Intermediate Precision

The ruggedness of the method was evaluated by performing the SVH bulk drug sample analysis in six replicates using two different columns, different instruments and different analysts on different days. This study was done to prove the compliance of different chromatograph (i.e. Shimadzu and Perkin Elmer) and analysts. The RSD of the results (n = 6) obtained with Shimadzu and Perkin Elmer were 4.2 and 6.1%, respectively. The overall RSD (n = 12) of this study for ECH content was 7.3%.

### Robustness

This study was performed by making small but deliberate variations in the method parameters. The effect of variation in carrier gas flow and column oven temperature on the ECH determination was studied. The results including system suitability test are presented in Tab. 2. Under all the variations, system suitability requirements (%RSD and S/N) were found to be well within the specified acceptance criteria.

## Discussion

The static HS-GC method was selected in this work because of the poor solubility of SVH drug substance in most of the organic and aqueous solvents. Also, the existing methods [10–14] involved derivatization procedures, adduct analysis, SPME fibers with thermal desorption, mass and electrochemical detectors for ECH determination only on clean sample matrices. The proposed HS-GC-FID method was validated for specificity, repeatability, linearity, accuracy, DL, QL, stability of analyte solution, intermediate precision and robustness. The HS incubation temperature and incubation time were identified as critical parameters and the optimum condition was finalized through extensive variation studies. Further, to demonstrate the robustness of the method, flow rate (± 10 %) and column oven initial temperature (± 10 %) were also studied and no significant variation in the ECH content was obtained. It is significant to note that ECH peak was well resolved and its specificity was proved by both HS-GC-FI and MS detection. Hence, retention time can be used as an identification tool for the ECH presence in SVH samples. The overall RSD of 7.3 % for intermediate precision demonstrated the ruggedness of the method. The DL, QL and recovery results indicated the accuracy and capability of the method to detect ECH at very low levels. The limitations and complexity associated with the reported methods [10–14] for drug substance analysis could be overcome by the proposed method as it is simple, utilizing direct HS extraction and most commonly used FI detection, without any derivatization and SPME fiber extraction. Moreover, the presently developed and validated method is cost-effective and simple to adopt at any pharmaceutical laboratory with moderate run time.

## Experimental

### Chemicals and reagents

Spectroscopy grade dimethyl sulfoxide (DMSO), N,N-dimethyl formamide (DMF) and 1-methyl-2-pyrrolidone (NMP) were procured from Merck (Mumbai, India). Analytical grade *N,N*-dimethyl acetamide (DMAc) was from s.d Fine Chem. Ltd (Mumbai, India). (±) Epichlorohydrin (99+%) was purchased from Sigma-Aldrich (Steinheim, Germany). Sevelamer HCl drug substance was obtained from Shasun Chemicals and Drugs Ltd., (Chennai, India).

### Chromatographic conditions

All experiments were performed on Shimadzu GC2010 Gas chromatograph equipped with AOC 5000 headspace sampler, flame ionization detector and GC Solution software (Shimadzu Corporation, Japan). A Clarus 500 Gas chromatograph having Turbomatrix 40 HS sampler, flame ionization detector with Totalchrom software (PerkinElmer, Shelton, USA) was used for intermediate precision and also for method development purpose. The specificity studies were carried out on a Shimadzu GC system coupled with quadrupole mass spectrometer (QP2010) and AOC5000 HS sampler (HS-GC-MS). GC-MS was carried out under electron impact (EI) ionization mode at 70 eV and interface, ion-source temperature were kept at 220 °C and 250 °C, respectively (Shimadzu Corporation, Japan). A Zebron ZB-Wax GC column, 60m length × 0.53mm ID × 1 μm film thickness was used (Phenomenex, Torrance, USA). The GC oven temperature program utilized an initial temperature of 50 °C, then increased at 7 °C per min to 220 °C and was held for 3 min at 220 °C. The injector was used in splitless mode. The temperature of injector and detector were kept at 150 °C and 230 °C, respectively. Helium was used as carrier gas at constant pressure mode (8.0 psi) equivalent to the initial flow rate of 6.0 mL/min. Injection volume of 1.0 mL for Shimadzu and injection time of 0.10 min for PerkinElmer instrument were used. HS conditions of both Shimadzu and PerkinElmer were presented in Tab. 3. Since, the HS hardware and injection techniques are different for these two instruments; some of the parameters of PerkinElmer were not applicable for Shimadzu and vice versa.

### Solution Preparation

Standard stock solution was prepared by dissolving 100 mg of ECH into a 100 mL glass volumetric flask containing 25 mL of DMSO (diluent), diluted to volume with diluent and 1.0 mL of this solution was diluted to 100 mL using diluent (10 μg/mL). System suitability solution was prepared by diluting 1.5 mL of standard stock solution into 100 mL and then, 3.0 mL of this solution was diluted to 10 mL using diluent (0.045 μg/mL). 2.0 mL of this solution was placed in a HS vial and sealed. Signal-to-noise ratio (S/N) for ECH peak from system suitability solution was evaluated with the acceptance criteria of not less than 3.0 (S/N). Standard solution was prepared by diluting 7.5 mL of standard stock solution into a 100 mL glass volumetric flask containing 25 mL of diluent and diluted to volume (0.75 μg/mL). 2.0 mL of this solution was placed in a HS vial and sealed. The relative standard deviation (%) for the ECH peak from six replicate injections of standard solution was evaluated with the acceptance criteria of not more than 5.0%. Sample preparation was made by placing 1000 mg of SVH sample in a HS vial containing 2.0 mL of diluent and sealed (500 mg/mL).

## Conclusion

The static HS-GC-FID method described in this investigation was proved to be an ideal tool for the determination of ECH in SVH polymeric drug substance at lower levels (< 0.3 μg/g) to comply with the ICH/FDA/EMEA regulatory requirement. Method validation data demonstrated that the developed method is sensitive as well as accurate for the estimation of ECH and robust to minor variations in the chromatographic parameters. The identity of ECH and specificity of the method were well established by both HS-GC-FID and HS-GC-MS detection. Hence, the proposed HS-GC-FID method can be employed conveniently in the pharmaceutical laboratory for the routine quality control of ECH in SVH drug substance.

## Figures and Tables

**Tab. 1.** Method validation data for ECH impurity

**Validation parameter**	**Results**			
	**ECH**			
Detection limit (μg/mL)	0.09			
Quantitation limit (μg/mL)	0.30			
Precision at QL (n=6, % R.S.D)	7.2			
Linearity				
Calibration range (μg/mL)	0.30–9.98			
Calibration Points	9			
Slope	1232			
Intercept	−87.6			
Coefficient of determination (r^2^)	0.999			
Accuracy				
Added (μg/mL)	0.300	0.751	1.501	1.802
Recovered (μg/mL)	0.275	0.704	1.443	1.740
% Recovery	91.7	93.7	96.1	96.6
% R.S.D (n=3)	6.4	8.4	3.3	4.7

**Tab. 2.** Robustness

**Variation**	**RT^a^**	**S/N**	**RSD (%)**	**ECH (μg/mL)**
No variation	10.10	3.9	3.0	1.451
Flow rate (+10%)	9.55	3.9	2.7	1.465
Flow rate (−10%)	10.68	3.5	2.1	1.493
Oven initial temperature (+10%)	9.50	4.2	0.8	1.332
Oven initial temperature (−10%)	10.67	3.6	1.8	1.540
RSD (%)				5.3

a retention time of ECH

**Tab. 3.** Head space analyzer conditions

**HS Parameter**	**Shimadzu**	**Perkin Elmer**
Incubation temperature	100 °C	100 °C
Incubation time	600 s	600 s
Transfer line temperature	n.a.	120 °C
Pressurization time	n.a.	4.0 min
Agitator speed	250 rpm	n.a.
Needle temperature	110 °C	110 °C
Pneumatic pressure	n.a.	15 psi

n.a. … not applicable
